# Applying augmented reality multimedia technology to construct a platform for translation and teaching system

**DOI:** 10.1016/j.heliyon.2024.e28700

**Published:** 2024-03-26

**Authors:** Yue Zhao, Qilin Wang

**Affiliations:** a School of Chinese Language and Literature, Liaoning Normal University, Dalian, 116021, China; b School of Information Science and Engineering, Dalian Polytechnic University, Dalian, 116034, China

**Keywords:** Multimedia, AR technology, Augmented reality, Computer, English translation

## Abstract

This paper investigates the integration of augmented reality (AR) technology into English translation teaching for college students, emphasizing the pivotal role of innovative teaching methods in enhancing students' translation skills and learning experiences. To address the issue of insufficient interest in English translation teaching, the paper initially assesses the purpose and significance of learning English translation through a questionnaire survey, elucidating challenges encountered in English language acquisition. Subsequently, adhering to AR principles, a teaching demonstration platform rooted in AR technology is conceived and developed, intricately aligned with English translation instruction. The platform serves as a solution to issues in English learning, such as inadequate course comprehension, low utilization of teaching resources, and instructors' lack of experience. The research culminates in the analysis of survey results, wherein the quantitative disparities in translation ability between students utilizing the research platform and those subjected to traditional teaching methods are examined. The findings underscore the positive impact of the AR-based research platform on improving students' translation proficiency. The AR platform heightens learners' engagement in the learning process, contributes to constructing a robust knowledge framework, and enhances overall learning outcomes. The platform offers educators opportunities to optimize experimental courses and elevate teaching standards. The paper's outcomes present novel pedagogical scenarios for learners, propose technical solutions for other technical disciplines and furnish a theoretical foundation and application model for a new generation of experimental demonstration platforms.

## 1 Introduction

With the rapid development of computer multimedia information technology, translation teaching research is facing unprecedented opportunities and challenges. Improving the effectiveness and quality of translation teaching with the assistance of computer multimedia information technology has become a topic of widespread concern in current education and translation circles. This paper aims to explore the related issues in this field, deeply analyze the potential role and value of computer multimedia technology in translation teaching, and contribute to enhancing the innovation and professionalism of translation instruction.

In recent years, computer multimedia technology has experienced rapid development, particularly with the application of Augmented Reality (AR) technology in education, which has gradually garnered widespread attention [1]. AR technology has demonstrated potential advantages and innovations, particularly in the education of children and young people [2]. However, while AR technology has made remarkable strides in the education of children and young people, its application research in college education is relatively limited [3]. This paper focuses on exploring the potential application of AR technology in college students' English translation teaching, aiming to provide new avenues and methods to enhance college students' translation ability and learning experience.

In traditional English translation teaching, students often encounter various challenges, such as memorizing basic knowledge, conducting grammatical analysis, and enhancing reading comprehension [4]. Despite the availability of numerous teaching methods and resources, the question of how to effectively stimulate students' interest in learning and enhance their learning outcomes remains a significant issue requiring thorough discussion [5]. This paper introduces AR technology into English translation teaching with the goal of enhancing students' motivation and engagement through innovative teaching methods, thereby improving their translation skills and overall learning experience [6]. The central focus of this paper lies in exploring how AR technology can be utilized to enrich students' learning experiences and bolster their translation skills within the context of English translation teaching for college students. To address this inquiry, the paper systematically investigates the potential applications of AR technology in English translation teaching and its impact on students' learning outcomes through surveys and experimental research. As an integral component of cross-cultural communication, English translation necessitates students to possess a strong language foundation, adept grammatical analysis skills, and proficient reading comprehension [7]. However, conventional teaching methods often struggle to cultivate students' interest in grammatical analysis, consequently impeding their ability to express themselves and think critically during the translation process [8,9]. To mitigate this issue, this paper innovatively proposes an English translation teaching solution centered around AR technology. By creating an immersive learning environment, students' interest in grammatical analysis is sparked, leading to enhancements in their translation and expressive capabilities.

Based on the aforementioned content, this paper contributes to research in the following ways. Firstly, it entails the design of a translation platform and questionnaire built upon AR technology. Secondly, it outlines the experimental design. Lastly, it employs a quantitative research methodology to comprehensively assess students' translation abilities by comparing the experimental group utilizing the AR translation platform with the control group that does not utilize the platform. The findings indicate a significant improvement in English two-way translation, reading comprehension, and grammar analysis within the experimental group, thereby demonstrating the effectiveness of AR technology in English translation teaching. This paper offers valuable insights and practical applications for innovatively integrating AR technology into English translation instruction for college students. It serves as a concrete case study for the implementation of AR technology in educational settings.

## 2 Literature review

AR technology, as an innovative and promising advancement, has witnessed significant development in the realms of mobile technology and computer vision in recent years. It adeptly integrates virtual information with the physical world, leveraging multimedia, 3D modeling, real-time tracking and registration, intelligent interaction, and sensing technologies to enable computer-generated text, images, music, video, and other elements to interact seamlessly with the real environment, thus delivering users an immersive experience [10]. Jang et al. (2021) discussed how AR technology facilitates the interaction of digital information within the physical environment via mobile devices, ushering in a new learning paradigm for learners [11]. Furthermore, AR seamlessly merges virtual elements with the real-world setting, offering users a fresh perceptual encounter [12]. Fueled by contemporary advancements in mobile technology and visual computing, AR technology has garnered increasing attention and application, effectively bridging the gap between the virtual and real worlds.

The interactive approach facilitated by AR presents educators with a novel teaching method, fostering the creation of an autonomous learning environment in the most natural and interactive manner [13,14]. AR has the capability to simulate non-existent objects, enabling users to visualize and interact with virtual entities in the physical realm. This functionality offers educators a novel means of presenting learning materials and establishing an environment conducive to independent exploration [15]. AR's capacity to depict abstract content is particularly illuminating for educational purposes, underscoring its significant potential for development and application within the educational sphere. While the most prevalent use of AR applications remains in entertainment, educational applications are still in their experimental stages, offering intuitive content but often lacking sufficient interactivity [16]. Presently, AR education applications suffer from inadequate content distribution and subject education, and often lack active engagement from both teachers and students [17]. Globally, numerous research teams have undertaken extensive investigations into key AR technologies, such as the 3D modeling research by Pujiastuti and Haryadi (2020) [18] and the virtual behavior interaction research by Ashley-Welbeck and Vlachopoulos (2020) [19], yielding fruitful outcomes and affirming the popularity of AR educational applications among students. While Chinese students currently demonstrate a high level of engagement with AR applications, international students have limited exposure to AR educational applications.

A virtual learning environment built upon AR technology represents a novel educational approach, yet its characteristics resonate with several educational theories. For instance, behaviorism posits that learning occurs through the connection between stimuli and responses, with stimuli triggering responses [20]. Batista et al. (2020) highlighted that within the AR virtual learning environment, learners engage with the surroundings, swiftly receive feedback, and make decisions for subsequent actions, thereby establishing links between knowledge and responses [21]. This environment offers a diverse range of tools and interactive spaces, fostering learners' autonomy in shaping the learning process. This pedagogical approach aligns with the concept of “bringing the laboratory into the classroom,” as advocated by Fernández-Enríquez and Delgado-Martín (2020) [22], and corresponds with the theoretical notion of learning as a genuine real-life experience proposed by Shadiev and Yang (2020) [23].

Much of the research on AR technology's application in education worldwide is empirical, with action research being predominant [24,25]. Scholars on the global stage have observed operational scenarios utilizing display devices and projection screens [26]. AR technology's enhanced capability to visually represent complex teaching materials has proven effective in reducing both physical and metaphorical distance between educators and learners, thereby promoting interaction and facilitating a deeper understanding of educational content. In the Chinese context, Gu et al. (2022) leveraged the Internet as the foundational platform for instruction, establishing a service system tailored to students in need of educational resources [27]. The primary aim is to develop an online distance learning system that facilitates remote education for students efficiently.

In summary, international research on AR technology's application in education is still nascent and largely empirical. The aforementioned analysis underscores the widespread use of AR technology in practical teaching activities. However, due to disciplinary constraints, the integration of AR in translation teaching remains relatively uncommon. Presently, English major courses often lack engagement, making it challenging for students to maintain interest in English translation. Therefore, exploring how AR technology can enhance the effectiveness of translation teaching holds significant importance.

## 3 Methodology

The issues within English translation teaching are identified and analyzed based on students' actual experiences through the administration of questionnaires, particularly focusing on attitudes and challenges encountered in English translation courses. Subsequently, AR technology is employed to develop an English translation teaching framework. The efficacy of this approach is evaluated through a comparative analysis with traditional methods. Finally, recommendations are formulated for universities and colleges regarding English translation teaching, drawing from the current findings and feedback obtained from students.

A. The present situation of English translation teaching
(1) Questionnaire design

Currently, the majority of college English teachers possess extensive translation experience, enabling them to effectively impart their knowledge and skills to students, thereby offering valuable support.

The questionnaire survey is designed to gather information and data from college students, covering several key aspects:

The objectives and motivations behind pursuing an English translation major. Challenges encountered during translation learning. Perspectives on the multimedia teaching model. Understanding of and attitudes towards the integration of AR applications. Feedback on the experience of utilizing AR-based English translation courses. The survey targets second-year students, both Chinese and international. A total of 328 questionnaires were distributed, of which 305 were returned. After excluding invalid responses, 282 questionnaires were considered eligible for analysis. The gender distribution among participants was approximately 54% male and 46% female, with Chinese and international students represented at 53% and 47%, respectively.

Statistical analysis was conducted on the responses, with a significance level (p) exceeding 0.05 indicating no statistically significant disparity between genders. This suggests that gender-related factors have minimal influence on the survey outcomes.

**Fig. 1:**
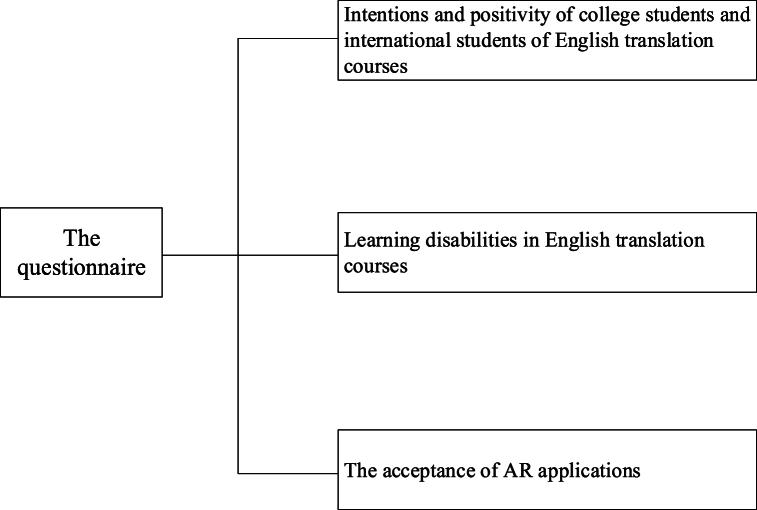
Structure of the questionnaire.

The questionnaire comprises three parts, as depicted in Fig. 1. The first part explores students' intentions and attitudes towards English courses, the second part gathers data on learning difficulties encountered in English translation courses, and the third part assesses students' acceptance of AR applications. It employs various question types, including multiple-choice and sequencing questions.

In the first part, the answer choices range from “totally agree” to “strongly disagree.” In the second part, the options range from “always” to “not at all."

Fig. 1 illustrates that the questionnaire's scale design predominantly consists of three main sections:

The first section delves into students' willingness and enthusiasm towards the English course, aiming to understand their attitudes, interests, and learning motivations. Specific inquiries include students' expectations for English learning, comprehension of course objectives, and career aspirations in English translation. To comprehensively capture students' perspectives, various question types are utilized, including open-ended queries, multiple-choice items, and scale questions. The second section focuses on gathering data regarding students' learning obstacles in the English translation course, with the goal of identifying potential challenges and difficulties they may face. Specific issues encompass challenges encountered during translation, language barriers, and skill disparities. This section collects students' real-life experiences in translation learning through a combination of multiple-choice questions and open-ended queries to gain insights into their learning challenges. The third section investigates students' acceptance of AR applications, seeking to evaluate their awareness and receptiveness to AR technology within the context of the English translation course. Specific inquiries address students' understanding of AR applications, perceptions of the teaching efficacy of AR, and interest in utilizing AR for English translation learning. Multiple-choice questions and scale questions are systematically employed to assess students' attitudes and opinions regarding AR technology.

(2) Data statistic

The results and experimental data were subjected to statistical analysis using SPSS 19.0 statistical software. The experimental outcomes underwent testing for normal variance homogeneity and were expressed as “mean ± standard deviation.” Additionally, the independent sample *t*-test was employed to compare data collected from the two groups, considering both homogeneity and heterogeneity of variance. Furthermore, One-way Analysis of Variance (ANOVA) was utilized to compare sample means among multiple groups. The results were presented in Excel tables and charts.

In conducting an independent sample *t*-test, it is essential to first consider whether the variances are homogenous or heterogeneous. The homogeneity test of variance determines whether the variances of the two groups are equal, which is a crucial condition for the *t*-test hypothesis. If homogeneity of variance is confirmed, the homogeneity of variance sample *t*-test can be applied. Conversely, if homogeneity of variance is not established, the variance sample *t*-test is necessary, taking into account the situation of unequal variances. In both tests, a significance level of α = 0.05 is set to determine whether a significant difference exists.

The homogeneity test of variance employs the Levene test, which is based on the variance of two groups. If the result of the Levene test is significant (p < 0.05), it indicates that variance heterogeneity is established, and the variance sample *t*-test is necessary. Otherwise, if the result of the Levene test is not significant, the homogeneity of variance sample *t*-test can be applied. Using these methods, data between different groups are compared to analyze the application effect of AR technology in the field of education, as well as the differences between traditional methods and AR methods. This analysis helps to verify the effectiveness and feasibility of the proposed method.

Based on the above content, the questionnaire data analysis steps in this paper are outlined as follows:

Step 1 The experimental results and data are statistically analyzed using SPSS 25.0 statistical software.

Step 2 The normal variance homogeneity test is utilized to assess the experimental results, represented as “mean standard deviation."

Step 3 An independent sample *t*-test is conducted to compare the differences between the two groups of data. In the case of homogeneity of variance, the independent sample *t*-test of homogeneity variance is employed. For non-homogeneous variance, the independent sample *t*-test of non-homogeneous variance is applied.

Step 4 One-way analysis of variance is employed to compare the sample mean values between multiple sets of data, with results presented in Excel tables and charts.

Step 5 Multiple regression analysis, including correlation analysis, is performed using mathematical models to investigate the relationship between curriculum management and interaction and student performance and satisfaction.

B. System design and implementation
(1) Overall design of ar-based teaching material implementation algorithm
1) AR Technology

AR technology is a technique that overlays computer-generated virtual information onto the real world in real-time. Through AR technology, users can perceive virtual elements via visual, auditory, or other sensory means, and these elements interact with the real environment. The aim of AR technology is to create an enhanced, integrated perceptual experience in the real world by organically combining virtual and real elements. This technology provides a richer, immersive user experience and has a wide range of applications, including gaming, healthcare, industry, retail, and more. In AR technology, computer-generated information can be presented in the form of three-dimensional images, text, audio, and other formats, offering users diverse perceptual input for real-time interaction with virtual content. The development of AR technology has opened up new possibilities for creating more immersive and interactive real-world experiences, fostering innovation and progress across various industries.

2) Platform Architecture Design

**Fig. 2:**
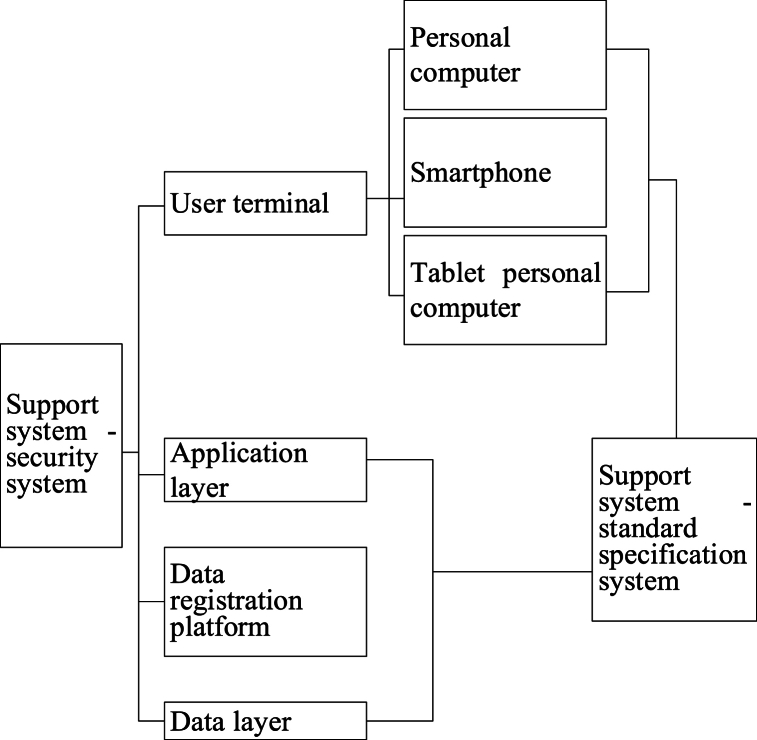
The overall architecture of the teaching demonstration platform.

Failure to achieve the objectives of experimental teaching may result if the instructor solely demonstrates, excluding students from conducting independent experiments. Therefore, it becomes imperative to incorporate a virtual operational skills training component through an experimental demonstration platform. This addresses equipment deficiencies within educational institutions and enhances students' proficiency in practical skills. In response to these challenges, the experimental demonstration platform integrates advanced AR technology with the distinctive features of translation teaching courses and the existing issues encountered in translation technology courses. This integration aims to capture students' attention, improve learning efficiency, and foster the development of their cognitive abilities and practical skills. The architecture of the experimental demonstration platform is designed to consist of three main layers: an application layer (comprising the experimental demonstration platform, teaching organization platform, and material management platform), a data registration layer, and a data layer, as illustrated in Fig. 2. Fig. 2 also highlights the “Use terminal” connected with each block diagram, featuring three-terminal devices at the same level: “Personal computer,” “Smartphone,” and “Tablet personal computer.” These terminal devices serve as user access points, offering students various access methods for convenient utilization of platform functions based on individual preferences and equipment selections. 10.13039/100014337Furthermore, Fig. 2 illustrates the interconnection between the application layer and the “supporting system,” which includes a “safety system” and a “standard specification system.” These auxiliary systems provide essential support to facilitate the seamless operation and comprehensive data management of the entire experimental demonstration platform. The lines depicted in Fig. 2 serve to delineate the interaction, dependency, or information flow relationships between distinct components as represented by different block diagrams.

**Fig. 3:**
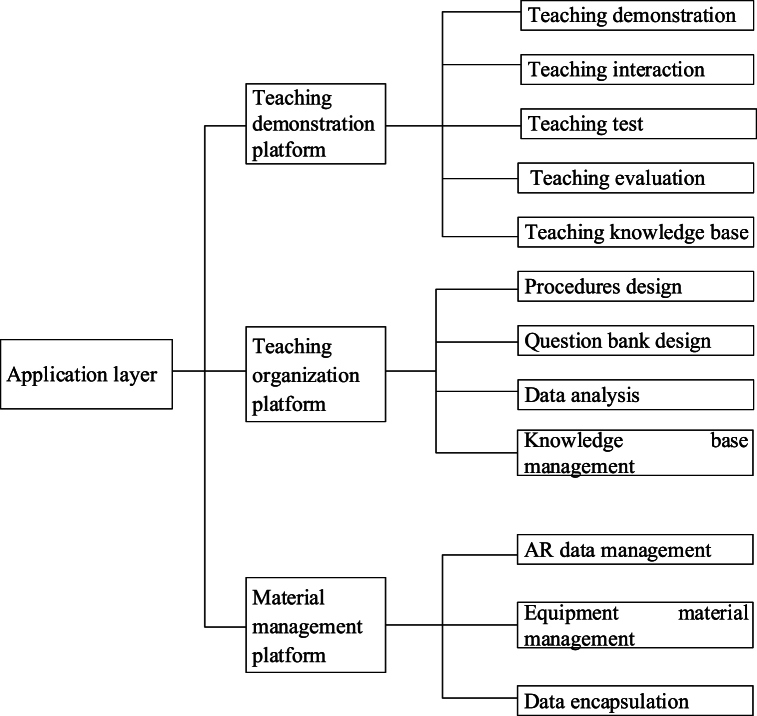
The structure of the application layer.= 2 \* GB3 ②The platform server serves as a repository for various types of data and provides data services via datasets. Platform managers are tasked with maintaining extensive data, databases, and servers. The constant evolution of data information poses challenges for the application layer to fully and directly integrate data resources. Hence, the necessity arises for a data registration platform to collect and manipulate data, facilitating resource sharing. Specifically, the structure of the data registration platform is shown in Fig. 4.

= 1 \* GB3 ①The application layer is a crucial component of the experimental demonstration platform, serving as the client interface with diverse and multifunctional capabilities. It acts as the client-side carrier of the platform, facilitating the visualization and interaction of computer-generated virtual elements through an AR technology-based display platform. This integration seamlessly combines virtual content with the real world, offering students a comprehensive learning experience. Its primary functions encompass demonstration, interaction, testing, evaluation, and database management. Firstly, within the application layer, the demonstration platform stands out as a primary tool for students to achieve learning objectives and enhance learning effectiveness. Students can intuitively engage with virtual elements through this platform, gaining a deeper understanding of instructional content and reinforcing knowledge mastery. Secondly, the interactive features empower students to engage in real-time interaction with virtual elements, thereby elevating their sense of participation and interest in the learning process. Educational organizers utilize the application layer to design teaching workflows, conduct demonstrations, design question banks, perform data analysis, and manage knowledge repositories through the educational organization platform. This provides educators with powerful tools to flexibly meet diverse teaching needs and ensure the efficient progress of the teaching process. Finally, system administrators operating within the application layer are responsible for managing the experimental demonstration platform, AR data, and equipment materials. Their duties include platform maintenance and updates, ensuring the system's proper functioning, and overseeing the management of data and equipment related to AR technology. The specific structure of the application layer is depicted in Fig. 3.

**Fig. 4:**
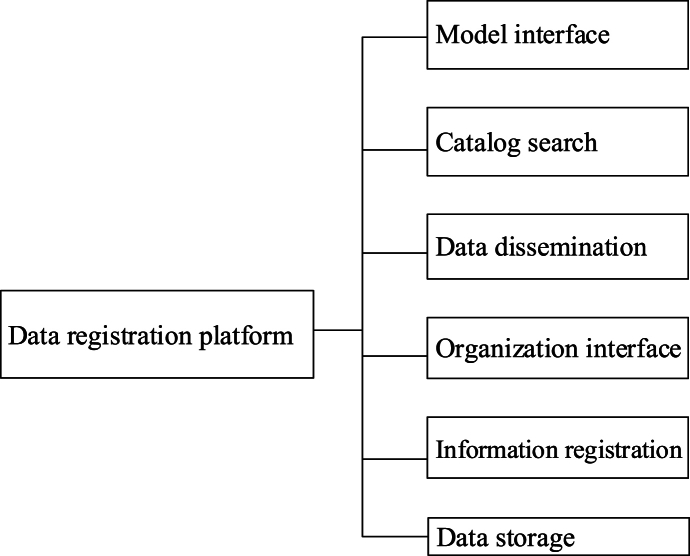
The structure of the data registration platform.= 3 \* GB3 ③The data layer serves to store and classify various types of data, particularly complex data associated with the demonstration platform. Within the AR experimental demonstration platform, the data layer encompasses user data, experimental model data, process data, timeline logic data, evolution model data, and evaluation system data. Specifically, the structure of the data layer is shown in Fig. 5.

**Fig. 5:**
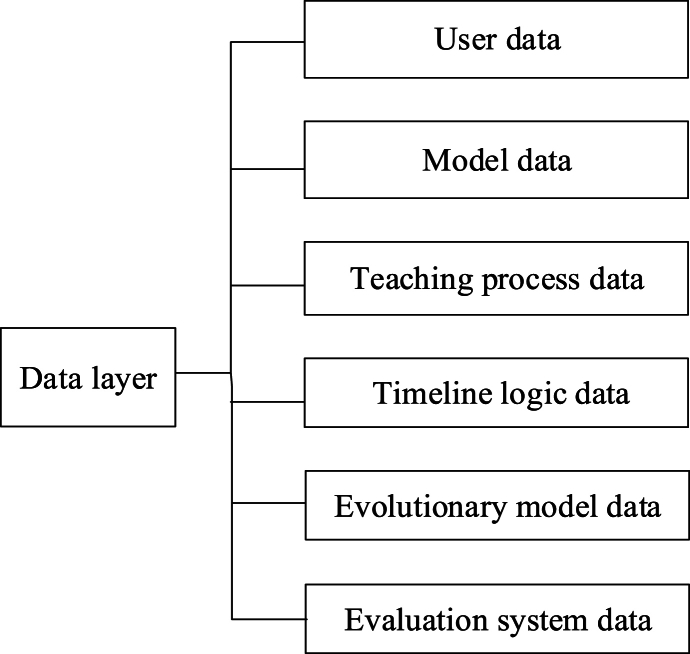
The structure of the data layer.

The Mobile AR system, supported by mobile devices, wireless networks, and local registration technology, enhances collaborative and contextual learning through computer simulation technology. It offers convenience, interactivity, contextuality, connectivity, and personalization. While some scholars have raised concerns about handheld mobile devices potentially distracting students and increasing the likelihood of task interruptions [28], AR systems can counteract this by detecting students' current locations and working statuses. Task reminders and waiting options can be issued promptly to promote students' concentration. These embedded reminders reduce the likelihood of task interruptions, thereby maintaining students' focus. Additionally, if a student's network-connected mobile device is involved in face-to-face interaction, its social interactivity is further enhanced.

(2) Functional module design

In the functional module design section of this paper, each functional module within the application layer is discussed in detail, emphasizing its design and function. The purpose of this section is to clearly outline the functional levels of the teaching demonstration platform and the roles and functions of each module in achieving the overall goal.

1) Management of teaching materials: Within the teaching demonstration platform, the material management platform is accessible to the material administrator for managing experimental materials and AR data. The administrator can categorize materials by type (such as model, video, picture, and text) and perform various management tasks including addition, modification, and deletion. Regarding AR data management, the material administrator oversees the production and modification of AR data. AR materials, including properties and interactive actions, are created using the AR editor.
2) Experimental teaching organization: This module is tailored for teachers. On the teaching organization platform, teachers have the capability to design courses (both theoretical and experimental), create question banks, and manage knowledge bases. Teachers develop teaching content, strategies, scenarios, and procedures in alignment with the curriculum. Subsequently, they select the necessary teaching materials, which may include AR materials with interactive features. During the teaching process, testing is a crucial component. Following tests, the platform provides feedback data to the teachers, conducts data analysis, and evaluates the class comprehensively both formatively and summatively. Teachers can then make appropriate adjustments to courses based on the evaluation results.
3) Teaching demonstration platform: Firstly, students are required to access the login homepage and obtain initial qualification to access the teaching demonstration platform. Subsequently, students must review and acknowledge the platform's usage rules before utilizing or engaging with the materials available on it. Serving as the central link, the teaching demonstration platform allows students to select practical courses based on their learning progression. However, due to current limitations in AR technology, students must first utilize mobile terminals to scan the AR identification map before AR materials can be displayed on monitors for real-time interaction. Upon completion of the courses, students undergo testing to assess their knowledge mastery, and the system provides feedback on the learning evaluation, allowing students to review and learn again if necessary.
4) Realization of teaching demonstration platform: The realization of an English translation teaching platform based on AR involves a complex technical process encompassing various key components and technical elements. Firstly, effective application of AR technology necessitates high-performance 3D modeling and rendering technology, as well as real-time tracking and positioning technology. Advanced 3D modeling tools are utilized to overlay virtual objects onto real-world objects, enabling students to interact with virtual objects in the real world through real-time tracking and positioning technology, thereby providing a realistic and immersive experience. Secondly, intelligent interactive technology plays a pivotal role in the platform's functionality. Various interactive methods, such as gesture recognition, voice recognition, and touchscreen interaction, are implemented to enable students to interact freely with virtual objects, thereby enhancing their sense of participation and promoting active learning and exploration. Additionally, the platform incorporates data analysis and evaluation functionalities. Interactive data from students on the platform are collected, and a data analysis algorithm evaluates their learning progress. These data feedback not only aid teachers in understanding students' performance but also provide the basis for adjusting courses and teaching strategies to meet students' learning needs. In practical application, this AR-based teaching platform serves not only as a technical tool but also as a powerful resource for improving students' English language skills. Through interaction with virtual objects, students gain deeper understanding of key concepts and skills in English translation. They can simulate real-life scenarios in the virtual environment through practical operations, thereby enhancing their practical application abilities. Such experiential learning fosters students' self-confidence, encourages more active engagement in the learning process, and enhances their proficiency in the English language.
5) The management mode and resource utilization of the AR platform knowledge base enable teachers and administrators to create and update knowledge base contents using specialized editing tools. This includes uploading and managing various resources such as 3D models, videos, pictures, and text. Teachers have the flexibility to add new content based on teaching needs or modify existing content to align with curriculum objectives and teaching progress. The knowledge base is organized and classified to facilitate convenient access and use by students. Resources are categorized according to topics, course units, or specific teaching scenarios, providing targeted AR experiences. This organization aids students in locating relevant resources for their learning needs. Students access these resources through the AR platform, selecting suitable ones according to their learning progress and requirements. For specific teaching activities, teachers can assign related AR experience tasks, prompting students to utilize platform resources to complete assignments. The knowledge base encompasses a wealth of real AR experience resources, including real-scene simulations and demonstrations obtained through 3D modeling, video recording, and photo collection, presenting real-world situations. This management approach ensures that the platform offers diverse, practical, and effective AR experiences for students. It enhances their understanding of learning content and facilitates application in practical contexts. Overall, this method of knowledge base management contributes to providing students with enhanced learning experiences, promoting deeper comprehension, and encouraging practical application of acquired knowledge.

**Fig. 6:**
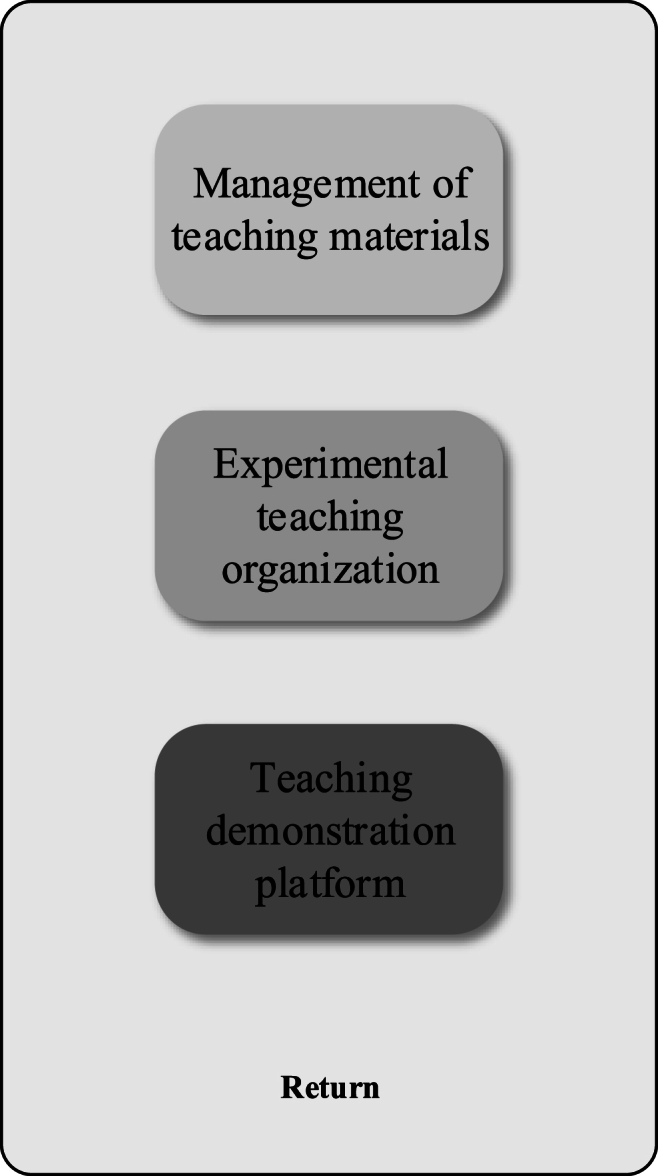
Platform interface design.(3)Database design

Expanding on the preceding content, this paper delves into the platform interface design of the system. Platform interface design involves the strategic organization and meticulous structuring of the application layer within the experimental demonstration platform. Its primary objective is to craft an intuitive, user-friendly, and efficient operational interface, enabling students, educational organizers, and system administrators to effortlessly and enjoyably utilize the platform's diverse functions. The platform interface design emphasizes the arrangement of elements, interaction methods, graphic presentation, and user experience to ensure swift comprehension and operation of the platform, thereby augmenting learning and teaching effectiveness. Through clear design, the platform interface visually showcases functions such as demonstration, interaction, testing, evaluation, and database management, furnishing users with a seamless and intuitive virtual learning environment. The interface design of the platform is shown in Fig. 6.

On the teaching demonstration platform, data are categorized into two main types: structured data and unstructured data.

Structured data, also known as row data, is organized within a relational database on the teaching demonstration platform. The following data items are identified through functional analysis of the platform: Student information, including ID number, name, gender, and class. Teacher information, including teacher number, name, and class. Textbook information, including version, name, credits, and chapters. Grade information, including test chapters, student number, name, gender, and score. AR model information, including chapter, number, name, and 3D model.

Unstructured data encompasses video, audio, pictures, and graphics information. These diverse data types require classification and integration for effective use. Videos are dynamic and time-efficient teaching resources, offering abundant knowledge and rich materials. They are utilized for case teaching, closely integrating with real society to present teaching contents clearly and realistically, thus enhancing teaching efficiency. In contrast, graphic data is characterized by compactness and accessibility. It offers unique pedagogical advantages due to its reduced size. By integrating graphics with textual materials, students gain perceptual resources and intuitive impressions, facilitating deeper comprehension of key learning points and aiding in information extraction from extensive content. Graphic data's visual impact allows for the portrayal of intricate concepts and information, capturing students' interest and attention. Moreover, graphic data promotes intuitive understanding among students, enhancing their ability to retain and apply acquired knowledge effectively.

## 4 Experimental design and results

A The intentions and positivity of college students in english translation study

Overall, the investigation of students' learning situations in English translation is divided into two levels. The first level involves assessing students' attitudes towards English translation learning and identifying any challenges they face in English translation courses. The second level focuses on evaluating students' abilities in English translation. Students' attitudes towards English translation learning play a crucial role in determining their learning motivation. Active motivation enables students to better understand English translation knowledge and actively engage in translation tasks, thereby enhancing their English thinking and translation abilities.

**Fig. 7:**
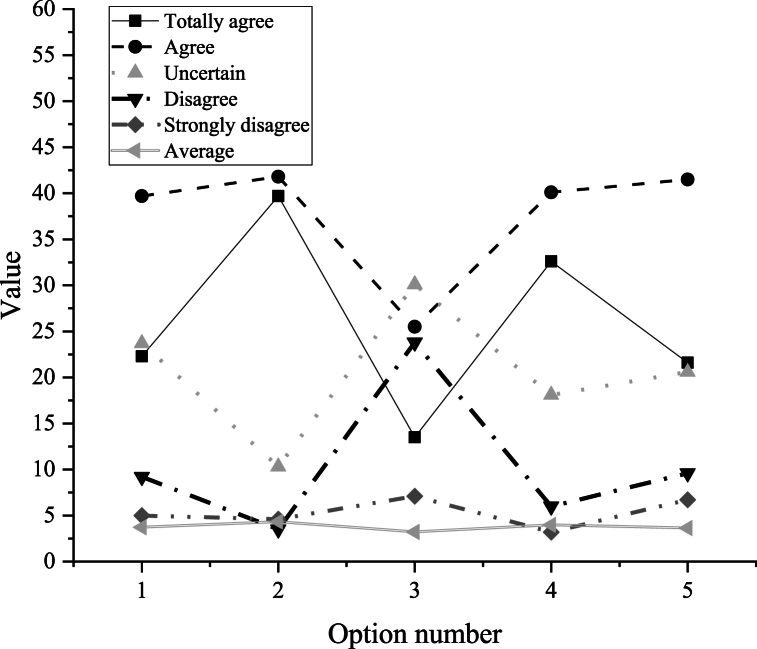
The intentions and positivity of college students in English translation courses.

In Fig. 7, the numbers 1–5 on the abscissa represent various aspects related to the Translation course, including its level of interest, relevance to daily life, ease of learning, the effectiveness of multimedia courseware in arousing interest, and overall satisfaction with the current English translation teaching situation.

Fig. 7 illustrates that college students generally hold positive attitudes toward English translation courses, as indicated by the subtotal average of 3.77. However, specific survey data highlight some noteworthy findings. Regarding question 1, the majority of students find English translation courses interesting, with 26% strongly agreeing and 37.7% agreeing. Conversely, only a small percentage express disinterest, with 5.3% strongly disagreeing.

Furthermore, question 2 reveals an average score of 4.32, indicating that students generally perceive translation courses as closely connected to daily life, with 81.5% totally agreeing or agreeing. A minority of students, comprising 3.5% disagreeing and 4.6% strongly disagreeing, consider translation unrelated to daily life. These findings suggest that most students can integrate English knowledge into their daily routines, providing a conducive environment for designing English translation teaching practices.

However, questions 3 and 4 highlight a challenge: while a small portion of students find English translation easy to learn, the majority perceive it as difficult. Despite their interest, students encounter challenges in learning English. Further investigation into the cognitive obstacles encountered by college students in English translation courses is warranted.

Overall, question 5 garnered an average score of 3.63, indicating that a majority of students are satisfied with current English translation courses (63.1% totally agree or agree). However, a significant percentage express uncertainty (20.6%), while a minority express dissatisfaction (disagree and strongly disagree: 16.3%). These results suggest that current English translation teaching in colleges and universities effectively enhances students' translation skills, but there is room for further improvement.

B. The obstacles encountered by college students in english translation learning

**Fig. 8:**
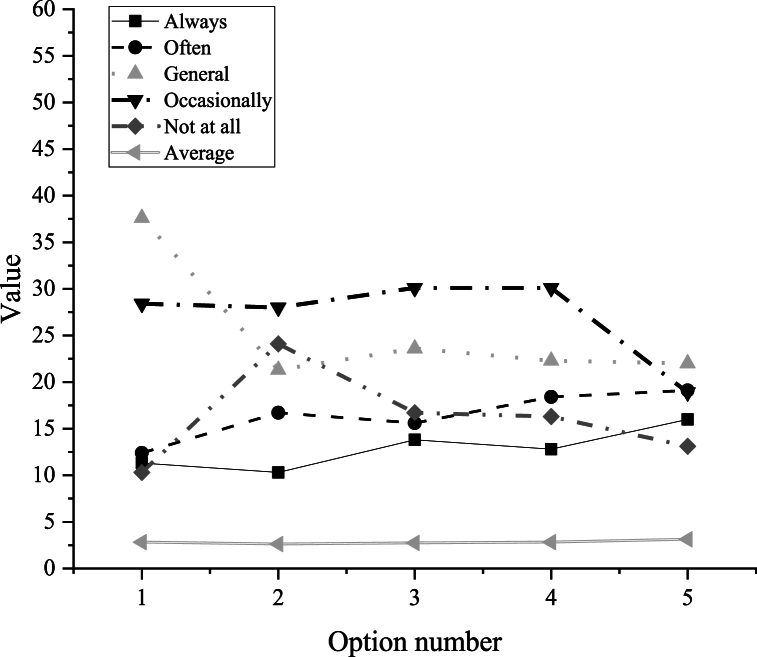
Obstacles encountered by college students in English translation learning.C.Mainstream ar education applications used by college students

Fig. 8 proposes investigating the challenges faced by college students in English translation across five dimensions. The first dimension pertains to the ability to memorize fundamental English knowledge, which is crucial for meeting the teaching objectives of English translation courses. Question 1 reveals that the distribution of students facing memory obstacles is concentrated at two levels: always (11.3%) and not at all (10.3%). However, the majority of students encounter difficulties in memorizing fundamental knowledge, with responses indicating occasional, frequent, or general difficulties (78.4%). While many memory-related issues are resolved during learning, some persist.

The subsequent four dimensions are contextualized within the university's cultivation of English proficiency. These dimensions include proficiency in translation usage and spelling, competence in spatial thinking within an English context, proficiency in comprehensive analysis during translation, and proficiency in practical operational skills in translation.

Among these dimensions, question 5 receives the highest average score of 3.12, while question 2 obtains the lowest score of 2.62. This data suggests that, in developing English translation proficiency, students demonstrate competency in addressing a majority of translation challenges through their disciplinary skills. The relatively consistent scores across various subjects indicate a balanced distribution of disciplinary competencies among students. In Fig. 8, the numbers 1–5 on the abscissa represent various challenges, including poor memory of English basic knowledge, inability to form a three-dimensional space image in the mind, difficulty in reading English and filling in blanks with words, lack of comprehensive analysis skills for articles, and difficulty in solving translation problems with open practice characteristics.

**Fig. 9:**
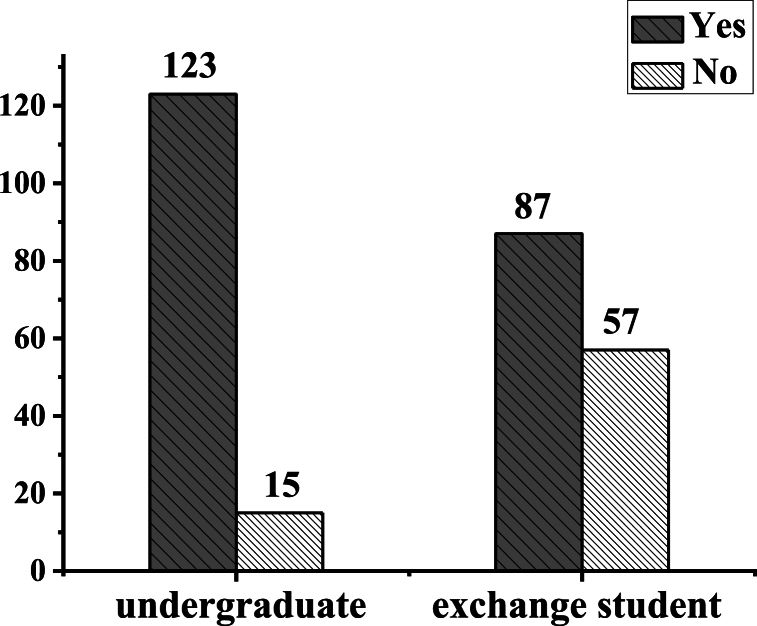
Survey results of whether the students have ever used an AR application.DComparison of the differences of translation ability between the group using ar translation platform and the traditional teaching group.

Fig. 9 illustrates the survey results regarding students' usage of AR applications. According to statistical data pertaining to the question, “Have you ever used an AR application (such as Youdao Dictionary, Sky Guide AR, Wikitude, Pokémon GO, HoloAR, FxCamera, and other applications)," it is revealed that nearly three-quarters of the surveyed students (74.5%) have utilized AR applications. Of note, the proportion of Chinese students who have used AR applications (58.6%) exceeds that of international students (41.4%), indicating a higher acceptance of M-AR applications among Chinese students.

This experiment employed a group comparison method to validate the constructed AR translation platform. Initially, 120 students majoring in English at a specific school were chosen. These students were then divided into experimental and control groups, with each group comprising 60 students. Selection for the experimental group was based on students' acceptance of new technology, academic performance, and interest in translation. This ensured that experimental group students had a foundational understanding of English translation and were capable of effectively utilizing mobile devices for learning. Students were randomly assigned to the experimental group, where they utilized the AR translation platform. For the control group, selection criteria were based on academic performance and interest in translation. These students were randomly assigned to the group that did not utilize the AR translation platform.

This paper examines the differences in translation proficiency between two groups of students: those who utilized the AR translation platform (experimental group) and those who received traditional teaching without the platform (control group). The study spans one semester, lasting a total of 15 weeks, for both the experimental and control groups. At the end of the semester, students from both groups undergo an assessment to evaluate their translation capabilities. The evaluation includes two-way translation exercises between Chinese and English (10 points), reading comprehension (10 points), and grammar analysis (10 points). These assessments are conducted and graded by a substitute teacher. The average scores and standard deviations for both groups are presented in Table 1.

**Table 1:** Average score and standard deviation results.

Test item	The average value of the experimental group	The standard deviation of the experimental group	Average value of control group	The standard deviation of the control group
English two-way translation	8.5	1.2	7.8	1.5
Reading comprehension	9	1	8.2	1.2
Grammatical analysis	8.8	1.3	7.5	1.6

In Table 1, the score of the experimental group is higher than that of the control group, which preliminarily shows that the translation teaching effect of this translation teaching platform is good. The results of the Levene test are shown in Table 2, including the variance of each test and the P value of the Levene test.

**Table 2:** Results of LEVENE test.

Test item	Experimental group variance	Control group variance	Levene test p-value
English two-way translation	1.44	2.25	0.127
Reading comprehension	1	1.44	0.359
Grammatical analysis	1.69	2.56	0.168

According to the results of the Levene test, the P value of the Levene test of all test items is greater than 0.05, which means that the null hypothesis cannot be rejected. That is, the variance of the two groups in each test is equal.

Next, because the homogeneity of variance is established, the independent sample *t*-test of homogeneity of variance is used to compare whether there is a significant difference in the average score between the two groups. The results are shown in Table 3.

**Table 3:** T-test results of variance homogeneous independent samples.

Test item	Average score of the experimental group	The average score of the control group	The standard deviation of the experimental group	The standard deviation of the control group	T value	Freedom	P value
English two-way translation	8.5	7.8	1.2	1.5	2.23	118	0.027
Reading comprehension	9.2	8.3	0.8	1.2	3.16	118	0.002
Grammatical analysis	9	8.2	1.3	1.4	1.98	118	0.05

According to the results of the *t*-test, it is evident that the average score of the experimental group is significantly higher than that of the control group (p **＜**0.05). However, no significant difference in average scores is observed between the experimental and control groups in the grammatical analysis test (p ＞ 0.05). This lack of significant difference in the grammatical analysis test results may be attributed to the nature of syntactic analysis falling within the domain of traditional grammar learning. It requires a relatively high level of proficiency in students' grammar foundations and logical reasoning abilities. The AR translation platform might not provide a significant advantage in this aspect, as it primarily focuses on visual and practical learning experiences. Students using the AR platform may prioritize these aspects, potentially overlooking the theoretical study of grammar rules. A comprehensive analysis of the experimental results suggests that the influence of the AR translation platform on improving students' translation ability involves various factors. Firstly, the AR translation platform may play a crucial role in stimulating students' interest in learning. By introducing innovative technology and interactive learning experiences, the AR platform may enhance students' interest in translation learning. This heightened interest may lead to increased engagement in the learning process and a greater willingness to explore and attempt translation tasks, thereby enhancing their learning motivation. Improved learning motivation, in turn, can positively affect students' learning performance. Increased interest and positive motivation can encourage students to delve deeper into course content, explore related knowledge more extensively, and interact more actively with learning materials. This positive learning attitude may have a favorable impact on students' translation ability, as they are more likely to invest sufficient time and energy into understanding and applying translation skills.

To further enhance the effectiveness of AR translation platforms in grammar analysis teaching, this paper proposes several strategies. Firstly, more practical cases and exercises for grammar analysis should be incorporated into the platform. These cases should cover varying levels of difficulty, ranging from basic grammatical structures to complex sentence analysis, to cater to the diverse learning needs of students. Secondly, an immediate feedback mechanism should be introduced to provide students with real-time error correction and suggestions during grammar analysis exercises. This feature will assist students in improving and refining their grammar analysis abilities promptly. Additionally, the integration of natural language processing technology and AR technology can be utilized to develop more intelligent auxiliary tools for grammar analysis. These tools will enable students to gain a deeper understanding of sentence structures and grammar rules. Furthermore, students should be encouraged to actively utilize the grammar analysis tools provided by the platform during the translation process. This practice will help cultivate their ability to independently analyze and resolve grammar issues, thus better equipping them to handle actual translation tasks confidently. By implementing these strategies, the effectiveness of the AR translation platform in grammar analysis teaching can be significantly improved. Students will be better prepared to tackle complex language analysis and translation challenges with confidence.

E. The relationship between curriculum management, interaction, and student performance and satisfaction

The questionnaire survey analysis shows the results of some curriculum management, interaction student performance and satisfaction scores in Table 4.

**Table 4:** Part of the curriculum management, interaction, and student performance and satisfaction score results.

student serial number	Curriculum management	Interaction	Student performance	Satisfaction
1	4.5	3.8	80	4.2
2	3.7	4.2	75	3.9
3	4.2	3.9	85	4.5
4	3.9	4.0	78	4.1
5	4.1	3.6	82	4.3
6	4.7	4.5	90	4.8
7	3.8	3.7	77	3.7
8	4.2	4.3	85	4.6
9	4.0	3.9	79	4.2
10	4.3	4.1	88	4.4
average score	4.16	3.9	81.1	4.1
standard deviation	0.29	0.215	5.032	0.192

In order to explore the relationship between curriculum management, interaction, student performance, and satisfaction, this paper uses a multiple regression model to analyze the questionnaire data in depth. The final multiple regression model is as follows.

BX=β0+β1×KCGL+β2×HD+ε

Student performance BX is the dependent variable, representing the performance level of students. Curriculum management (KCGL) and interaction (HD) are independent variables representing the curriculum management and interaction level. β0 represents the intercept, signifying students' baseline performance when both curriculum management and interaction variables are zero. β1 and β2 are regression coefficients, elucidating the influence of curriculum management and interaction variables on student performance. ε is an error term representing a random error the model cannot explain.

In addition, the correlation analysis results of the four variables, using the data analysis software, are shown in Table 5.

**Table 5:** The correlation analysis results between curriculum management, interaction, and student performance and satisfaction.

	Curriculum management (kcgl)	Interaction (hd)	Student performance (bx)	Satisfaction
Curriculum management (kcgl)	1	0.75	0.65	0.85
Interaction (hd)	0.75	1	0.72	0.78
Student performance (bx)	0.65	0.72	1	0.88
Satisfaction	0.85	0.78	0.88	1

Table 5 shows that the correlation coefficient between curriculum management and interaction is 0.75, indicating a strong positive correlation between them. The correlation coefficient between curriculum management and student performance is 0.65, indicating a positive correlation between them, but the relationship is weak. This shows a certain degree of positive correlation between curriculum management and student performance. However, this relationship is not as strong as the relationship between curriculum management and interaction. This experimental phenomenon may suggest that in this educational environment, the manner of course design and management has a relatively minor impact on student performance. In contrast, interactions among students may be a key determinant of academic outcomes. Perhaps teaching methods that emphasize fostering positive interaction among students could play a more significant role in improving student performance. The correlation coefficient between interaction and student performance is 0.72, indicating a strong positive correlation between them. This means that in this data set, there is a significant positive correlation between the level of interaction and student performance. The improvement of the interaction level is related to improving student performance. The correlation coefficients between satisfaction and the other three variables, which are 0.85, 0.78, and 0.88, respectively, are high. The data indicate that there is a strong positive correlation between satisfaction and curriculum management, interaction, and student performance.

## 5 Discussion

Based on the experimental results of this paper, the following in-depth discussions are made:

Firstly, in exploring the differences in translation ability between students using the AR translation platform and traditional teaching students, it is found that college students have a positive attitude towards English translation courses. Most students find English translation courses interesting, and only a few students lack interest in the course. This indicates that students' overall interest in English translation courses is high.

Secondly, when exploring the obstacles encountered by college students in English translation learning, it is found that the proportion of students with memory impairment is low, and most students struggle with memorizing basic knowledge. This suggests that students can generally overcome most memory problems in the learning process, but some difficulties still persist.

Next, regarding the comparison of translation ability, the average score of the experimental group is significantly higher than that of the control group (P＜0.05). However, in the grammatical analysis test, there is no significant difference in the average score between the experimental group and the control group.

Finally, according to the results of variable correlation analysis, as the level of curriculum management improves, there is also a relatively high trend in the level of interaction. Furthermore, the improvement in interaction level is associated with enhanced student performance. Additionally, the correlation coefficients between satisfaction and curriculum management, interaction, and student performance are high, indicating a close positive correlation between satisfaction and these three factors. Students with higher satisfaction levels generally perform better in curriculum management, interaction, and student performance.

## 6 Conclusion

In the context of translation teaching under computer multimedia information technology, it becomes particularly important to investigate the learning attitudes, abilities, and their relationship with the application of AR technology among English translation students. This paper delves into the analysis of university students' performance in learning English translation, exploring students' attitudes toward translation courses, encountered challenges, and the impact of the AR translation platform on enhancing students' translation abilities.

According to survey results, most students consider English translation courses interesting; they can realize the close connection between translation courses and daily life. The various changes in English translation courses can arouse students' original interests, which is an innate advantage of English translation courses. However, the experimental data and questionnaire results indicate that most students' interests in English translation courses significantly decrease as practical activities are implemented. As students’ interests decrease, they may lose the internal motivation to learn English, leading to a decline in subjective initiative and ability to learn English translation. The AR translation platform can stimulate students' learning motivation by enhancing their interest in learning, thus affecting their translation ability.

Globally, research on AR applications in education focuses on the current situations, technologies, market conditions, and brand marketing of AR technology. The primary users of AR applications are also concentrated among children and youngsters, with fewer college AR users. Here, a user experience model of a university M-AR translation application is built according to the characteristics of English translation courses in colleges and universities, students’ learning situations, theories of user experience, and the research method of user experience in the AR field. The M-AR technology is applied to the translation courses, and the model is modified to guide the M-AR applications in English translation courses. The system is implemented according to the characteristics and needs of translation students.

Innovatively, AR technology is applied to translation teaching, which is significant for the informatization development of translation teaching [29]. AR teaching resources can create unique learning situations, help students construct knowledge systems, present 3D information, and display information via multiple media. Additionally, AR can break temporal and spatial limitations, enabling users to access teaching resources anytime and anywhere, which is conducive to improving the learning experience and enhancing teaching tools.

AR and other immersive learning media can provide students with a sense of presence and enhance their intuition, making them more focused [30,31]. Firstly, students perceive the environment provided by AR as unique yet consistent. This sense of presence enhances students' perceptions of the learning community. Secondly, an AR system fosters students' intuition by offering immediate feedback and voice or non-speech prompts. Intuition is significant for cultivating emotional value in learning, and AR can integrate students, virtual elements or information, and specific characteristics into a unified world. Therefore, AR has great potential in cultivating students’ intuition [32,33]. Finally, immersive media, such as AR, offers students an immersive experience, which is a subjective feeling that a person has in a comprehensive and real experience. An immersive environment enables the transformation of learning content into problems and environments in the real world.

However, only a few components of English translation courses are selected for primary application design. While the AR translation system performs well in two-way translation and reading comprehension, its educational capability in grammar analysis requires improvement. The research findings of this paper are largely aligned with the original objective. The experimental subjects mainly consist of student groups from a specific university, which may not fully represent students from other schools or educational backgrounds. The limitation of the sample could impact the universality and generalization of the research outcomes. Further enhancements are necessary in the application's functional flow, content, and interaction design to address all challenges encountered by English translation majors. The research outcomes serve as specific references for English translation teaching. However, as AR technology and equipment continue to evolve, future application designs will encounter numerous challenges. To address these concerns, this paper aims to expand the research sample by including students from a broader range of schools and educational backgrounds to enhance the generalizability of the findings. Additionally, it seeks to conduct a more comprehensive analysis of students' usage and feedback on the AR translation system regarding syntactic analysis, aiming to pinpoint specific deficiencies and implement corresponding improvements. Furthermore, the paper suggests incorporating advanced natural language processing technologies in subsequent translation platforms to offer more accurate and practical translation assistance tools. The contribution of this paper lies in exploring the application of AR technology in translation teaching, innovating teaching methods, validating its effectiveness through experiments, and proposing relevant teaching strategies. This holds significant practical significance for promoting the advancement of modern educational technology and enhancing teaching practices.

## Fundings

Project of the 14th Five Year Plan for Education Science in Liaoning Province: Research on Improving the Emergency Comfort Speech Ability of Liaoning University Teachers (Project Number: JG21DB304).

## Ethics statement

.

## Ethical approval

The paper was conducted in accordance with the Declaration of Helsinki and approved by the Institutional Review Board of Liaoning Normal University (protocol code 2021.3823323, approval date 2021.7.22).

Written informed consent was obtained from the patients for their anonymized information to be published in this article.

## CRediT authorship contribution statement

**Yue Zhao:** Writing – original draft, Methodology, Formal analysis, Data curation, Conceptualization. **Qilin Wang:** Writing – review & editing, Visualization, Validation, Supervision, Software.

## Declaration of competing interest

The authors declare that they have no known competing financial interests or personal relationships that could have appeared to influence the work reported in this paper.

## Figures and Tables

**Table:** 

Question	Totally agree	Agree	Uncertain	Disagree	Strongly disagree
The translation course is interesting.					
The translation is closely related to daily life.					
Translation course is easy to learn.					
Multimedia courseware can arouse my interest.					
I am satisfied with the current English translation teaching situation.					

**Table:** 

Options	Always	Often	General	Occasionally	Not at all
I have a poor memory of basic English knowledge.					
I can't understand the thinking habits of foreigners.					
There is always a lack of idiomatic expression in translation.					
I don't know how to analyze the material comprehensively.					
I feel that it is difficult to solve translation problems with open practice characteristics.					

**Table:** 

Yes	No

## Appendix 1 Questionnaire

### Esteemed Classmates, Greetings! We extend our sincere gratitude for your participation in our survey. The primary objective of this questionnaire is to ascertain your academic progress in the translation class and discern your stance regarding the integration of augmented reality technology within the translation curriculum. We kindly request you to meticulously review each question and respond candidly based on your academic experiences at the institution. It is important to note that there are no right or wrong answers to any of the questions, and we encourage you to respond genuinely without undue deliberation. Your input should be reflective of your actual experiences and personal viewpoints. Thank you for your cooperation

Please read each question carefully, choose the answer that fits your situation, and tick “√.”

1. Your grade is A. Freshman. B. Sophomore. C. Junior. D. Senior
2. Your gender is: A. Female B. Male
3. The attitudes toward translation courses are described below. Please tick your answer below.

4.Please tick the answer below to describe the difficulties you encountered in the learning process of the translation course

5 Have you ever used an augmented reality APP (e.g., Youdao dictionary camera pickup, QQ eye dictionary, Sky Guide AR, Wikitude, Holo AR special effects camera, and other games and applications)?.
